# Native circulating *Brucella melitensis* lineages causing a brucellosis epidemic in Qinghai, China

**DOI:** 10.3389/fmicb.2023.1233686

**Published:** 2023-09-20

**Authors:** Hongmei Xue, Zhijun Zhao, Jianling Wang, Li Ma, Jiquan Li, Xuxin Yang, Lingling Ren, Liqing Xu, Zhiguo Liu, Zhenjun Li

**Affiliations:** ^1^Department of Brucellosis Prevention and Control, Qinghai Institute for Endemic Disease Prevention and Control, Xining, Qinghai, China; ^2^National Institute for Communicable Disease Control and Prevention, Chinese Center for Disease Control and Prevention, Beijing, China; ^3^Vocational and Technical College, Inner Mongolia Agricultural University, Baotou, China

**Keywords:** *Brucella melitensis*, whole-genome sequencing, MLVA, WGS–SNP, phylogenetic analysis

## Abstract

Since 2010, the cases and incidences of human brucellosis have been increasing annually in Qinghai (QH) Province. Molecular epidemiology and phylogenetic analyses of strains from this region are crucial to better understand the transmission of the disease and the evolutionary patterns of *Brucella* strains. In this study, classical bio-typing assay, multilocus variable-number tandem repeat analysis, and the whole-genome sequencing–single-nucleotide polymorphism approach were used to illustrate the epidemiological and evolutionary patterns of *Brucella melitensis*. A total of 54 *B. melitensis* bv. 3 strains were isolated and molecularly characterized, with all strains belonging to the East Mediterranean lineages. Cross-regional transmission events (i.e., between counties) were caused by common sources of infection, suggesting that predominant circulating genotypes are endemic in different regions. Strengthening surveillance in animal brucellosis and controlling infected animals’ cross-border movement are necessary. Two strains isolated from humans and marmots were clustered in the same sub-clade, implying the possible existence of direct and/or indirect contact between sheep (and goats) and wildlife (marmots), but this needs to be verified by further investigations. The global-scale phylogenetic analysis indicated that 54 strains sorted into six subclades, four of which formed independent lineages, suggesting that the increase in the incidence rate of human brucellosis may be caused by local circulating lineages. Further strengthening the serology and pathogen surveillance of animals (wildlife) and humans will contribute to an in-depth understanding of the transmission chain of human brucellosis in this region.

## Introduction

Brucellosis (Malta fever) is a globally distributed zoonotic disease. It has severe adverse effects on public health and the agriculture field, including population, livestock, and wildlife health. Since David Bruce first isolated the organism (*Micrococcus melitensis*) in 1887 (Godfroid et al., 2005), 12 species have been identified in the genus *Brucella*, that is, six classical and six novel species (Occhialini et al., 2022). *Brucella melitensis* is the most common pathogenic species in humans and animals, followed by *Brucella abortus* and *Brucella suis* (Xavier et al., 2010). Brucellosis can be transmitted to humans through the food chain or by direct/indirect contact with infected animals, such as consumption of animal raw milk and meat products or aerosol transmission (Minas et al., 2007). For centuries, brucellosis has been critically endangering human health and has led to substantial economic losses (Wareth, 2019). In some industrial countries, brucellosis has been effectively controlled, but it remains a serious public health risk to the majority of the population in developing areas. For example, in China, brucellosis is widely endemic in all 32 provinces (Rossetti et al., 2017).

Qinghai (QH) Province is located in northwestern China, and animal farming is the main source of income for the local population. Because of low development levels, poverty, and poor hygiene conditions, animal and human brucellosis is highly prevalent in this region (Ma et al., 2016). Although a comprehensive control plan for brucellosis was carried out during 2005–2010, which utilized the *Brucella* S2 vaccine for the immunization of ruminants combined with a serology test, and brucellosis-positive animals were eliminated, persistent funding is a great obstacle to control programs. Subsequently, the prevalence of human brucellosis increased annually in QH from 2005 to 2019 (Ma et al., 2020). Since 2010, the epidemic situation of human brucellosis has gradually become severe: the number of cases increased from 3 in 2010 to 756 in 2021, the incidence rate increased from 0.054/100,000 to 12.76/100,000 accordingly, and the affected geographic territory is currently expanding. Based on the national brucellosis surveillance sites, data indicate that the prevalence rate of human brucellosis was 3.35% (54/1,612) in 2019 and 4.77% (80/1,677) in 2020. However, the ability to tailor a cost-based brucellosis control program requires accurate and robust molecular typing tools to investigate the relationships between strains involved in common outbreaks and determine the source of infection and transmission routes (Pelerito et al., 2021). Generally, classical microbiological techniques allow researchers to obtain and bio-type the strains and facilitate molecular epidemiological investigations of the disease. The multilocus variable-number tandem repeat analysis (MLVA) has been used as the gold standard for genotyping *Brucella* strains, with the results combined with epidemiological data to investigate the relationships between the *Brucella* strains (Kiliç et al., 2011; Liu et al., 2017). Furthermore, single-nucleotide polymorphisms (SNPs) based on whole-genome sequencing have excellent power to discriminate strains and allow for the characterization of the phylogenetic relationships of strains from different scales (Janowicz et al., 2018; Abdel-Glil et al., 2022). Importantly, molecular typing tools can not only limit control costs and test time but also, improve the surveillance and evaluation of control measure effects. Therefore, classical bio-typing assay, MLVA, and whole-genome sequencing–SNP (WGS–SNP) were used to illustrate the species/biovars’ genetic diversity and the phylogeography pattern of *B. melitensis* from humans in QH to better assess the epidemiology profile and enhance brucellosis surveillance and control.

## Methods

### Strain source, identification, DNA isolation, MLST, and MLVA typing assay

A total of 54 *B. melitensis* strains were isolated and identified in the present study, of which 52 were recovered from humans, one from the liver of an aborted sheep fetus, and one from a marmot. All of the tested strains were isolated and identified according to the standard *Brucella* spp. bio-typing procedures (Yagupsky et al., 2019). The DNA of all 54 strains was isolated based on a two-step procedure: (1) the strains were heat inactivated at 80°C for 10 min, and (2) a QIAamp DNA kit (Qiagen, Heidelberg, Germany) was used to prepare the DNA of strains according to the manufacturer’s protocol. Following extraction, the harvested DNA from each strain was detected by agarose gel electrophoresis, and the DNA concentration was determined using a Qubit® 2.0 Fluorometer (Thermo Fisher Scientific, Waltham, MA, USA). MLST genotypes were deduced from WGS data using the PubMLST database (Jolley et al., 2018).^1^ The MLVA genotyping and data analysis of the strains (Supplementary Table S1) were performed as previously described (Liu et al., 2017).

### Genome sequence of *Brucella melitensis* strains

The genome sequencing strategy of strains was referenced in a previous study (Li et al., 2020). Briefly, all 54 *B. melitensis* strains were submitted for whole-genome draft sequencing, and the NEBNext® Ultra™ DNA Library Prep Kit for Illumina platform (New England Biolabs [NEB], Ipswich, MA, USA) was used to yield sequencing libraries according to the manufacturer’s specifications, as follows: the quality-tested extracted DNA was fragmented using the E210 Covaris instrument (Covaris, Inc., USA), and segments with approximately 350 bp in length were selected in a 3% agarose gel. The selected DNA fragments were then end-repaired, A-tailed, and ligated to Illumina-compatible adaptors (Bio Scientific, Austin, TX, USA) and then PCR-amplified using Illumina adapter-specific primers and Platinum Pfx DNA polymerase (Invitrogen), and the paired-end sequencing library was completed. Then, the draft genomic sequence of 54 strains was determined, and SOAPdenovo software v.2.04 (Li et al., 2010) was used to assemble and integrate good-quality paired reads into several scaffolds.

### SNP phylogenetic analysis of *Brucella melitensis* strains on local and global scales

WGS–SNP phylogenetic analysis of 54 *B. melitensis* strains was performed as previously reported (Li et al., 2020). Subsequently, phylogenetic analysis on the global scale of 133 strains was performed, of which 54 strains were from QH and 79 from GenBank (Supplementary Table S2), including 20 strains that were selected from five genotypes in a previous study, which are marked in red in (Supplementary Table 2) (Pisarenko et al., 2018), and 38 strains from China. The remaining 19 strains were from other countries (such as Italy, Pakistan, Egypt, Afghanistan, Albania, and Iran) with high incidence rates, and *B. melitensis* 16 M (Chromosome accession numbers: NC_003317.1 and NC_003318.1) was used as the reference genome (DelVecchio et al., 2002). Subsequently, the sample genomes were aligned to the reference genomes described above using Nucmer (Kurtz et al., 2004), and the SNP calling and filtering steps were performed using the “show-snps” application (a module of MUMmer with the parameter “-ClrTH”) from the MUMmer package (Kurtz et al., 2004). Finally, BLAST (Ye et al., 2006) and RepeatMasker software (Tarailo-Graovac and Chen, 2009) were used to filter SNPs located in repeated regions to obtain reliable SNPs. The nucleotide substitution rate of sequences was used to estimate the phylogenetic relationships of strains. The phylogenetic trees were generated using the maximum likelihood (PHYML) method, with a bootstrap number set to 1,000 with orthologous genes. The phylogenetic tree was further annotated with the top-level clusters identified using RhierBAPS programs via R packages (Tonkin-Hill et al., 2018).

## Results

### Bio-typing, geographic distribution, hosts, and isolated time of *Brucella melitensis*

**Figure 1:**
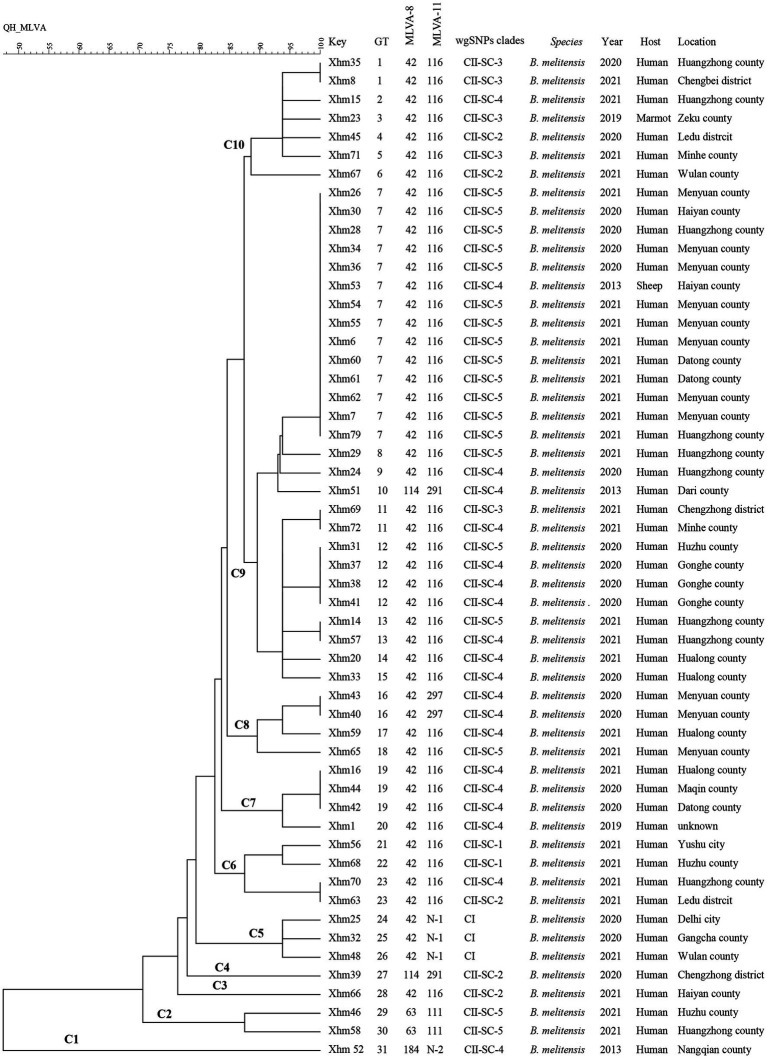
MLVA dendrogram of 54 strains from this study. The columns show the identification numbers (key), MLVA-16 genotypes (GT), panel 1 genotypes (MLVA-8) and MLVA-11 (panels 1 and 2A) genotypes, wgSNP clades, species-biovar, the year of isolation of the strains, host, and their geographic location. MLVA: multilocus variable-number tandem repeat analysis.

Based on the bio-typing approaches, 54 strains were identified, and all strains were identified as *B. melitensis* bv. 3 (Table 1). Furthermore, strains were distributed in all eight regions, with numbers ranging from 2 to 16 (Figure 1; Table 2), namely, 16 in Xining City, 11 in Haidong City, 15 in Haibei Prefecture, 1 in Huangnan Prefecture, 3 in Hainan Prefecture, 2 in Guoluo Prefecture, 2 in Yushu Prefecture, and 3 in Haixi Prefecture. Strains were isolated from three hosts (humans, domestics, and wildlife), namely, 52 humans, 1 sheep, and 1 marmot (Table 2). Moreover, 54 strains spanned the period 2013–2021, that is, 3 in 2013, 2 in 2019, 19 in 2020, and 30 in 2021.

**Table 1:** Bio-typing characteristics of 54 *Brucella melitensis* strains in this study.

Strains	Growth		Dye inhibition test		Monospecific serum		Phage lysis test			No.	Interpretation
	CO_2_	H_2_S	BF	TH	A	M	Tb	BK_2_	Wb		
BA	+	+	+	−	+	−	CL	CL	CL	1	BA bv. 1,544
BM	−	−	+	+	−	+	NL	CL	NL	1	BM bv. 1 16 M
BS	−	++	−	+	+	−	NL	CL	CL	1	MS bv. 11,330
Test strains	−	−	+	+	+	+	NL	CL	NL	54	BM bv. 3

BA, *Brucella abortus*; BM, *B. melitensis*; BS, *Brucella suis*; BF, basic fuchsin; TH, thionin; “+,” positive; “−,” negative; CL, confluent lysis; NL, no lysis.

**Table 2:** Area distribution and numbers of the 54 *Brucella melitensis* strains* in this study.

City	County/district (no.)	No. of strains
Xining City	Chengzhong District (2), Chengbei District (1), Huangzhong County (10), Datong County (3)	16
Haidong City	Ledu District (2), Minhe County (2), Huzhu County (3), Hualong County (4)	11
Haibei Prefecture	Haiyan County (3), Gangcha County (1), Menyuan County (11)	15
Huangnan Prefecture	Zeku County (1)	1
Hainan Prefecture	Gonghe County (3)	3
Guoluo Prefecture	Maqin County (1), Dari County (1)	2
Yushu Prefecture	Yushu City (1), Nangqian County (1)	2
Haixi Prefecture	Delhi City (1), Wulan County (2)	3

No., number of strains in each region.

* The location of one strain is unknown.

### MLST and MLVA genotypes of *Brucella melitensis* isolated from QH

In this study, MLST genotypes were deduced from WGS data using publicly available databases. All 53 *B. melitensis* strains were deduced as being the ST 8 genotype in both the 9-loci and 21-loci MLST approaches, and only one strain (XHM1) was identified as ST 68 (Supplementary Table S3).

Based on the MLVA assay, four MLVA-8 genotypes were identified, that is, 42 (*n* = 49), 63 (*n* = 2), 114 (*n* = 2), and 118 (*n* = 1), whereas six MLVA-11 genotypes were found, that is, four known [111 (*n* = 2), 116 (*n* = 44), 291 (*n* = 2), and 297 (*n* = 2)] and two newly identified (N1 (*n* = 3) and N2 (*n* = 1)), which are all single-locus variants to MLVA-11 genotype 116. These data indicated that all strains belonged to the Eastern Mediterranean lineage (Figure 1).

Based on the MLVA-16 analysis, 54 strains were sorted into 31 genotypes (GT1–31), of which eight genotypes (GT1, GT7, GT11–13, GT16, GT19, and GT23) were each shared by at least two strains (Figure 1). GT7 had the largest shared genotype, and it included 14 strains from different regions in 2013 (*n* = 1), 2020 (*n* = 4), and 2021 (*n* = 9), implying that a major genotype is endemic in these regions, and indicating a lack of control over the spread of this disease between regions. Moreover, XHM53 from GT7 was obtained from the liver of an aborted sheep fetus, and two strains (XHM7 and XHM62) in this shared genotype GT7 were isolated from a couple (husband and wife). Another two strains (XHM42 and XHM44) from the shared genotype GT19 were obtained from a family (father and son) who had a contact history with aborted lambs. These data suggest that each infection event was caused by a common source of infection. The remaining 23 genotypes were all singular, and each represented only one strain (Figure 1), suggesting epidemiologically unrelated and sporadic epidemic characteristics of human brucellosis. Remarkably, XHM23 (GT3) was obtained from a marmot blood sample collected in Zeku County (QH) that represented a unique MLVA genotype, but further genomic investigation is needed (Figure 1).

### SNP analysis and comparison with MLVA of *Brucella melitensis* strains

**Figure 2:**
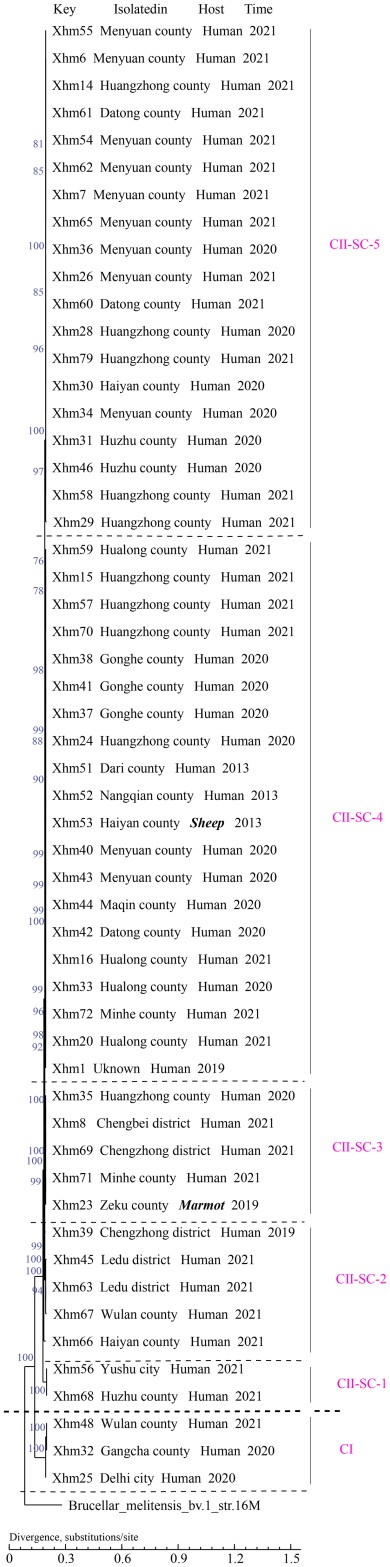
Phylogenetic tree of 54 strains from this study based on the WGS–SNP phylogenetic analysis. WGS–SNP, whole-genome sequencing–single-nucleotide polymorphism.

All 54 strains were divided into two clusters (I and II) based on the WGS-SNP phylogeny analysis (namely CI and CII), and CII was further sorted into five sub-clusters (CII SC1-5) (Figure 2). Furthermore, the strains with epidemiological links were grouped into the same sub-clusters; this result is consistent with that of the MLVA analysis, suggesting that these cases have a common source of infection. For example, strains from two shared MLVA genotypes with epidemiological links (GT7, XHM42, and XHM44; and GT19, XHM7, and XHM62) were sorted into the same sub-clusters (CII SC-4 and CII SC-5). Additionally, three shared MLVA genotypes (GT1, GT12, and GT16) were clustered into each of the same sub-clades: GT1 (XHM35 and XHM8) was clustered as CII SC-3; GT12 (XHM37, XHM38, and XHM41) clustered as CII SC-4; and GT16 (XHM40 and XHM43) clustered as CII SC-4 (Figure 1). Importantly, all clusters (and sub-clusters) comprised strains from different counties, which implied that multiple *B. melitensis* lineages were circulating in QH, causing the cross-regional human brucellosis epidemic.

### SNP phylogenetic analysis of *Brucella melitensis* strains on a global scale

**Figure 3:**
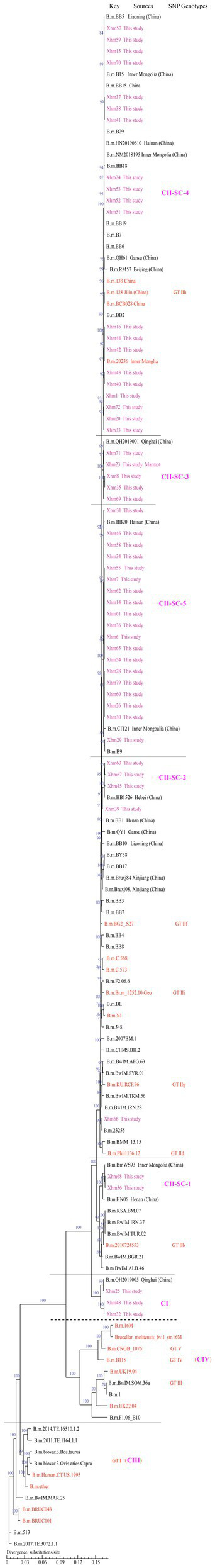
WGS–SNP phylogenetic analysis of 133 *Brucella melitensis* strains on a global scale. GT I–V is the same as previously described (Pisarenko et al., 2018) and marker with red, strains from the present study marked with pink, C I and C II from Figure 2 was used to code the clades. WGS–SNP, whole-genome sequencing–single-nucleotide polymorphism. The scale bar indicates the nucleotide sequence divergence.

To illustrate the phylogenetic profiles of strains in this study on a global scale, an SNP phylogenetic analysis of 132 *B. melitensis* was performed. The total number of SNPs found in all 132 *B. melitensis* strains ranged from 289 to 2,038 (Supplementary Table S4). These strains were categorized into four clades (CI–CIV) (Figure 3; Supplementary Figure S2; Supplementary Table S1). CI consists of three strains from the present study, CII is GTII, CIII is GTI, and CIV contains three SNP genotypes (GTIII–V), (Figure 3; Supplementary Figure S2). The phylogenetic clades were identified and verified using RhierBAPS programs, supporting the results of this study. Phylogenetic analysis indicated that all 54 strains belonged to the GT II lineage, which corresponds to the East Mediterranean lineages (Figure 1). Furthermore, two strains in QH-2 (XHM56 and XHM68) clustered into GT IIb, and 20 strains from CII-SC-4 clustered into GT IIh. Strains of three sub-clades (CII-SC-1, 4, and 5) clustered into the same sub-lineages with strains from Inner Mongolia, suggesting that there were epidemiological links between strains from Inner Mongolia and QH. The XHM23 isolated from the marmot (marked in bold) (QH-9) and B.m.QH2019001 from humans were clustered into the same sub-clades; both were previously isolated from QH, China. These data suggest that the two cases were caused by a common source of infection (Figure 1). Additionally, the strains in this study formed at least four independent clades (CI and CII-SC-3-5) (Figure 1), implying that these strains were the local-specific epidemic lineages, but further investigations are warranted.

## Discussion

In the present study, both classical bio-typing procedures and two molecular tools (MLVA and WGS–SNP) were used to characterize the *B. melitensis* strains from QH, China, to explore the molecular epidemiological relationship. The results of this work provide the crucial evidence necessary to formulate a targeted surveillance and control program. In the present study, the *B. melitensis* species was a predominant pathogen that was isolated from three different host species, and it was widely distributed. In particular, *B. melitensis* was isolated from samples from many hosts, including yaks, sheep, blue sheep, and Tibetan gazelle (Ma et al., 2016; Cao et al., 2018). These data revealed that there was a high diversity of natural reservoir hosts that allowed *B. melitensis* to continue circulating in this province. Indeed, animals are a natural reservoir host for zoonotic organisms and the majority of human brucellosis infections originate from animal hosts (Recht et al., 2020). Sheep and goats are optimal hosts for *B. melitensis*, and they can infect many different hosts such as cattle, swine, and deer (Liu et al., 2020). Thus, brucellosis control in regions with multiple existing hosts is a significant challenge. A targeted and comprehensive control program for infected sheep and goats should be implemented as a priority strategy to curb the spread of this disease to humans. Several further measures should be implemented, such as prohibiting the blind expansion of the breeding industry, strengthening the inspection and quarantine of animals for importation and exportation, widening the wildlife surveillance zone, improving the awareness of disease prevention among practitioners, and banning the circulation of sick animals.

In this present study, four MLVA-8 genotypes and six MLVA-11 genotypes were identified: 42 (MLVA-8) and 116 (MLVA-11) are predominant genotypes, accounting for 90.7% (49/54) and 81.5% (44/54), respectively. These data indicated that all strains were of Eastern Mediterranean lineage. With the use of MLVA-8, the majority of strains (84/105) were genotyped, and 42 were clustered into the East Mediterranean lineages (Jiang et al., 2011). Furthermore, MLVA-11 genotype 116 is a predominant circulating genotype in China, accounting for 69% (951/1382) (Zhu et al., 2020). Importantly, most *B. melitensis* strains belong to the East Mediterranean group, which comprises strains from Europe, the Middle East, and Asia (Vergnaud et al., 2018). MLVA genotype 116 is predominant in Asian countries (e.g., 86.8% in Kazakhstan) (Shevtsova et al., 2019). Furthermore, MLVA analysis data suggested that epidemiologically related characteristics of *B. melitensis* infection and cross-regional transmission events are caused by common sources of infection, suggesting that the predominant circulating genotype is endemic in different regions; this indicates a lack of control over infected animals’ movement and exchange between regions. In the Middle East, uncontrolled animal transportation through “open” borders is a main risk factor for brucellosis spread between some regions (Gwida et al., 2010). We hypothesize that the nomadic lifestyle (which often involves the consumption of raw milk) and production methods (e.g., homemade dairy products) in highland pastoral areas may have caused these infection events, and a detailed field survey is needed to verify the conclusion of this molecular investigation. A nomadic lifestyle may favor the spread of brucellosis among different animals and populations (Liu et al., 2022) because animals and people live in close contact. A 2018 report found that the consumption of raw milk from smuggled sick goats caused human brucellosis epidemics (25 cases) in Douz, Tunisia (Charaa et al., 2022). The purchase and consumption of cheese and milk from non-regulated sources are very common in specific communities among Israeli Arabs, with nearly 41 and 16.1% of respondents consuming cheese and milk, respectively, from non-regulated sources (Baron-Epel et al., 2018). Therefore, ruminant vaccination, control of cross-border animal movements, and control of non-regulated goat milk sales must be strengthened to prevent the spread of brucellosis.

WGS–SNP analysis has proved to be a robust molecular tool for illustrating the phylogenetic patterns of *Brucella* strains (Tan et al., 2015; Georgi et al., 2017). It demonstrated that multiple *B. melitensis* lineages were circulating in QH. Similarly, WGS–SNP phylogenetic analysis resolved Chinese *B. melitensis* strains into five clusters, reflecting the existence of multiple lineages (Sun et al., 2017). In addition, strains from humans and marmots were clustered in the same sub-clades, implying the possible existence of direct and/or indirect contact between sheep (and goats) and wildlife (marmots). Indeed, wildlife has a crucial role in the epidemiology of brucellosis in animals and humans (Galarce et al., 2021). Therefore, strengthening prevention, surveillance, and control of wildlife is recommended.

Global phylogenetic analysis indicated that 54 *B. melitensis* strains were clustered into genotype II (Pisarenko et al., 2018) and further divided into six sub-lineages, revealing the existence of multiple circulating lineages in QH. Additionally, many sub-lineages were shared by strains from this study and strains from Inner Mongolia, Hainan Province, Shandong Province, and Hebei Province, indicating that these strains have a potential phylogenetic relationship (Liu et al., 2020). The cross-border movement and transfer of animals between Côte d’Ivoire and Mali for grazing and/or trade have exacerbated the spread of brucellosis across the region (Oyetola et al., 2021). Therefore, genome sequencing in more strains and building a local genome database are necessary to improve the surveillance capacity and control the spread of brucellosis (Liu et al., 2023).

Although we obtained important insight in our investigation, some limitations are worth acknowledging. First, most strains in genome sequencing were obtained over a 2-year period, and more strains involved during a longer period could facilitate a more profound description of the brucellosis epidemiological profile of this region. Second, obtaining field epidemiological data in many cases is challenging. However, these data provide crucial auxiliary support for genome epidemiology.

## Conclusion

*Brucella melitensis* is a predominant species, and its distribution has been widespread in all nine regions in QH. *B. melitensis* strains belonged to the East Mediterranean lineages, and the human brucellosis epidemic in recent years was potentially caused by many native circulating lineages. Strengthening the genome sequencing of strains from a variety of host sources will facilitate the identification of transmission routes and determine potential ongoing outbreaks, which is vital for formulating targeted surveillance and countermeasures.

## Data availability statement

The datasets presented in this study can be found in online repositories. The names of the repository/repositories and accession number(s) can be found in the article/Supplementary material.

## Ethics statement

The research protocol was reviewed and approved by the Ethics Committees of the Institute for Endemic Disease Control and Prevention of Qinghai (No. 2022006). Informed consent was obtained from all patients. The studies were conducted in accordance with the local legislation and institutional requirements. The participants provided their written informed consent to participate in this study. The animal study was approved by the Ethics Committees of the Institute for Endemic Disease Control and Prevention of Qinghai (No. 2022006). Informed consent was obtained from all patients. The study was conducted in accordance with the local legislation and institutional requirements.

## Author contributions

HX performed the strain isolation, collection, and epidemiology data process. ZhiL and ZheL participated in the design of the study, analyzed the sequencing data, and drafted the manuscript. ZZ, JW, LM, JL, XY, and LR participated in the field epidemiology survey. ZhiL, ZheL, and LX critically reviewed the manuscript and managed the project. All authors contributed to the article and approved the submitted version.

## Funding

This work was supported by the National Natural Science Foundation of Qinghai, China (No. 81860588). The funders had no role in the study design, data collection and analysis, decision to publish, or preparation of the manuscript.

## Conflict of interest

The authors declare that the research was conducted in the absence of any commercial or financial relationships that could be construed as a potential conflict of interest.

## Publisher’s note

All claims expressed in this article are solely those of the authors and do not necessarily represent those of their affiliated organizations, or those of the publisher, the editors and the reviewers. Any product that may be evaluated in this article, or claim that may be made by its manufacturer, is not guaranteed or endorsed by the publisher.

## Footnotes

^1^ https://pubmlst.org/organisms/brucella-spp

## Abbreviations

WGS–SNP: whole-genome-sequencing–single-nucleotide polymorphism
MLVA: multilocus variable-number tandem repeat analysis
